# Validation of a bitmap of genes involved in cherry fruit cracking by digital PCR and qPCR, suitable for plant breeding

**DOI:** 10.1038/s41598-025-11006-w

**Published:** 2025-07-22

**Authors:** Marlene Santos, Alberto Gila-Navarro, Julia Weiss, Berta Gonçalves, Manuela Matos, Marcos Egea-Cortines

**Affiliations:** 1 Centre for Research and Technology of Agro-Environmental and Biological Sciences , CITAB, Inov4Agro, University of Trás-os-Montes and Alto Douro, UTAD, Quinta de Prados, 5000-801 Vila Real, Portugal; 2 Instituto de Biotecnología Vegetal, Universidad Politécnica de Cartagena Campus Muralla del Mar, Cartagena, 30202 Spain; 3 Department of Biology and Environment (DeBA), University of Trás-os- Montes e Alto Douro, Vila Real, 5000-801 Portugal; 4 Department of Genetics and Biotechnology (DGB), University of Trás-os- Montes e Alto Douro (UTAD), Vila Real, 5000-801 Portugal

**Keywords:** DPCR, Fruit cracking, Cherry, Cell wall metabolism, Wax biosynthesis, Bitmap, Plant breeding, Plant stress responses, Genetics, Plant sciences

## Abstract

Fruit cracking is a developmental defect depending on genetic and environmental conditions. Fruit cracking has a negative impact on quality and production. Fruits with cracking cannot be commercialized and enhance pathogen contaminations. Identifying genes as markers may help in breeding and post-harvest treatments. We compared qPCR and dPCR methods using a set of 16 genes that appear to be differentially expressed in the cherry varieties Sweatheart with low cracking and Burlat with high cracking indexes. Differences in absolute transcripts spanned across nearly three orders of magnitude. Overall qPCR and dPCR show a highly significant negative correlation of -0.90. The equation allowed converting Ct values to dPCR copy number. However, copy number in dPCR allow a direct comparison across experiments and transcriptomic analysis. The combination of *PaCER1*, *PaXTH*, *PaEXP1*, *PaEXP2*, *PaKCS6*, *PaWINA*, *PaWINB* and *PaCER3* as an expression bitmap can separate cherry fruits with low and high cracking phenotypes based on gene expression. Our results highlight the importance of the wax biosynthesis and cell wall metabolic pathways in susceptibility to fruit cracking. Furthermore, the newly identified bitmap may be useful to test in other locations and with different varieties.

**Supplementary Information:**

The online version contains supplementary material available at 10.1038/s41598-025-11006-w.

## Introduction

Fruit cracking is a developmental abnormality with a genetic component and susceptible to environmental conditions. It affects many fruits such as cherries, prunes, litchi, pomegranate or bananas, just to name a few^1–3^. The molecular mechanisms causing fruit cracking comprise a combination of changes in cuticle and cell wall metabolism, water and calcium transport, transcription factor activities and hormone responses^2^. Years of research in different plants have identified a list of differentially expressed genes that underlie the molecular mechanisms involved in the biological process of fruit cracking. Cracking indexes above 20–25% will cause unprofitable productions^4^. Fruit cracking has both genetic and environmental component^5^. Among the environmental factors influencing cracking in cherries are humidity, excessive rainfall during fruit development and high temperature^6^.

A significant issue in gene expression studies of agricultural and field biology is the effect of environmental cues and conditions on gene expression. Major players include light^7^ temperature^8^ or nutrients such as nitrogen or calcium^9,10^. Thus, having a clear picture of gene expression variation may help identify candidate genes for breeding and agricultural diagnostic. If we consider environmental effects, the variance in gene expression may hinder the comparison between experiments.

While genomes entail the information of a species, transcriptomes are the first layer of biological coordination. Changes in gene expression are responsible for developmental changes and adaptation to the environment. Traditionally transcriptome analysis started studying one gene at a time. The method of choice for single gene differential expression analysis is qPCR. It can be performed either with gene specific fluorescent probes or with a fluorescent dye intercalating between double stranded DNA molecules as they are synthesized^11,12^.

The SYBR green qPCR technology is a very well-established method to analyze changes in gene expression. Changes in gene expression quantification require either a calibration curve for each gene analyzed^13^ or a comparison against reference genes^14,15^. As qPCR tests DNA synthesis in every cycle, some relative quantification algorithms require the analysis of PCR efficiency^16–18^. Thus PCR primer design should be ideally customized to obtain maximum efficiency^19^. Most qPCR machines will give as output a Ct value, or cycle threshold. The Ct value is the cycle where fluorescent signal is higher than a given internal threshold.

In contrast, dPCR reads the accumulated fluorescence as a binary signal at the end of the reaction, thus the name digital^20^. Prior to fluorescence reading, template molecules are spread across a fixed number of nano-wells or partitions where each reaction takes place. Sample dilution has to be performed to obtain at least one third of empty partitions, to ensure that each well has one initial template molecule^21^. While qPCR results using SYBR green technology are usually given as Ct values, dPCR results are expressed as number of wells amplified per reaction. Thus, qPCR is usually performed as relative quantification assay while dPCR gives absolute quantification.

In this work we have compared qPCR vs. dPCR using a set of samples of sweet cherries with low and high cracking index^4,5^. We used a set of 14 genes previously described as differentially expressed in sweet cherry^22^. We also analysed two previously uncharacterized genes *PaEXP2* and *PaCYP78A9*. Gene expression levels across the dataset encompasses three orders of magnitude. The nested sample analysis of qPCR Ct values vs. log transformed dPCR counts showed a highly significant correlation of −0.907 with a p value < 2.2e-16. Our results indicate that dPCR has great potential to be used in agricultural and plant experiments where sample variation is high.

## Results

**Fig. 1:**
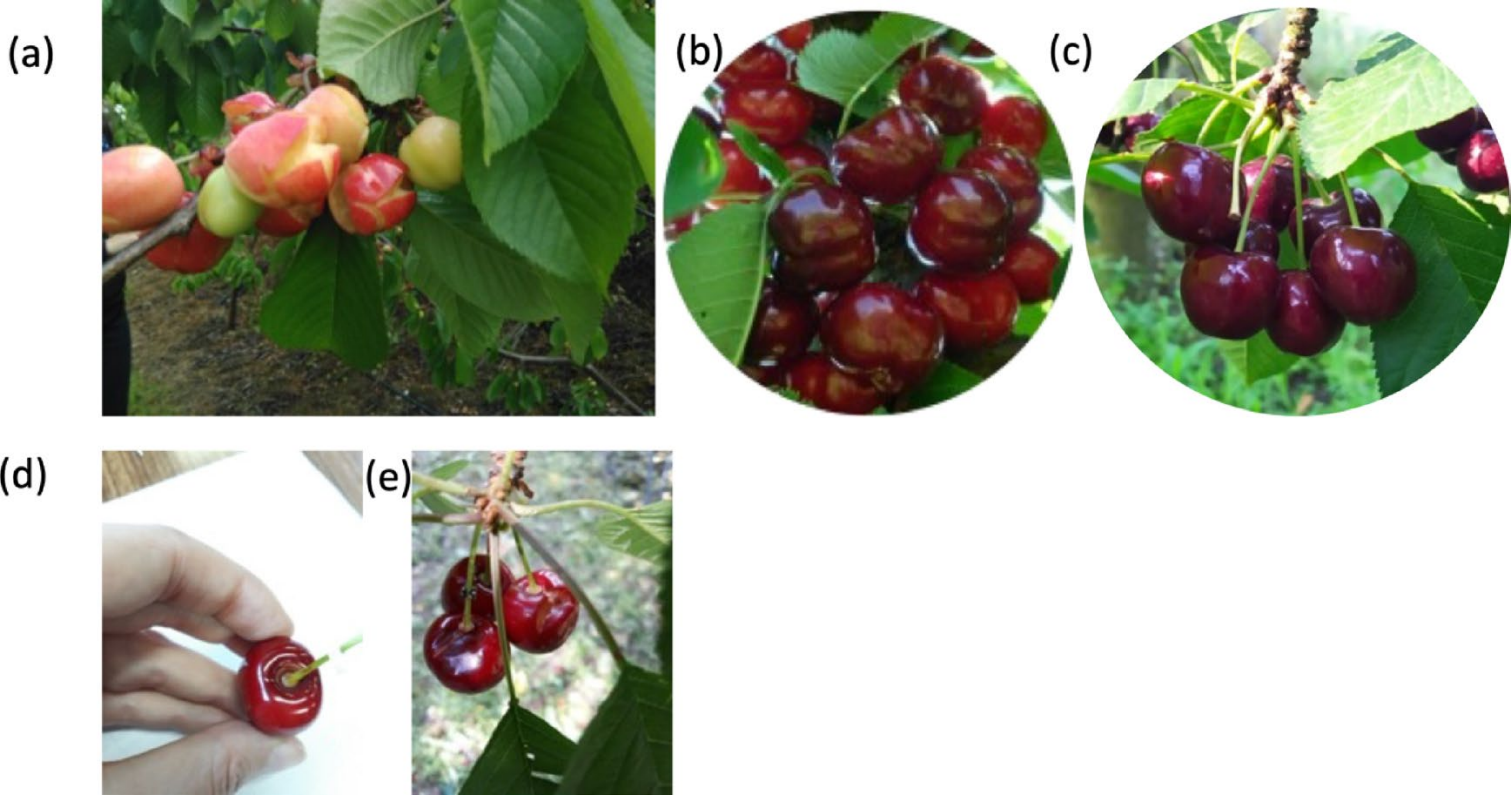
Fruits showing cracking damages in an orchard of Burlat variety (**a**). Healthy fruits in orchard of Burlat (**b**) and Sweatheart (**c**). Two typical cracking phenotypes at the pedicel (**d**) and across the pericarp (**e**).

Fruit cracking is easily spotted in orchards (Fig. 1), but can be readily induced by immersion in distilled water (see materials and methods).

### Analysis of gene expression by qPCR vs. transcripts number by dPCR

Differential gene expression was analyzed in a set of samples with low CI (*n* = 10) and high CI (*n* = 9). We used qPCR and dPCR to estimate gene expression. The gene with the highest expression in qPCR in both CI groups was *PaPIP1;4*. However according to the dPCR, the most expressed gene, also in both CI groups, was *PaXTH* (Tables 1 and 2). The genes with lowest expression were *PaLACS2* in low CI, and *PaWINA* in high CI according to qPCR. dPCR determined *PaWINA* to be the least expressed gene in both CI groups. Overall gene expression was higher in samples with high CI when considering raw values. Once normalized with *PaACT*, however, this tendency was inverted, and most genes were found to be under-expressed in high CI samples compared to low CI samples. The dPCR copies showed an up to 20-fold difference between some genes like *PaXTH*. However, qPCR derived Ct values showed differences of around 2 to 4 cycles between CI groups. The magnitude of the change in expression level can be understood in an easier way with dPCR since count data serves as a direct measurement in opposition to Ct values. The dPCR data has a very large SD as compared to qPCR. This is due to the logarithmic nature of Ct values (Supplementary Fig. 2) which reduces apparent variance (Tables 1 and 2).

**Table 1:** Quantification results of qPCR in high and low CI samples.

Genes	Mean Ct ± SD High CI	Mean Ct ± SD Low CI	*P*-value adjusted	Mean FC ± SD
PaAct	16.17 ± 0.38	20.57 ± 1.56	NA	NA
PaB-Gal	25.03 ± 1.04	27.3 ± 1.39	0.0004	0.26 ± 0.16
PaCer1	23.05 ± 1.33	24.99 ± 0.73	0.0019	0.26 ± 0.26
PaCer3	27.21 ± 0.59	26.99 ± 0.54	0.0001	0.04 ± 0.01
PaCYP79A9	24.37 ± 1.68	28.67 ± 2.32	0.7197	1.33 ± 1.1
PaEG	24.71 ± 1.95	25.47 ± 1.37	0.0037	0.23 ± 0.44
PaExp1	17.81 ± 1.12	20.84 ± 0.9	0.0213	0.51 ± 0.34
PaExp2	16.37 ± 0.86	19.78 ± 1.79	0.0147	0.61 ± 0.42
PaKCR1	21.45 ± 1.06	24.79 ± 1.16	0.0431	0.64 ± 0.49
PaKCS6	26.18 ± 0.87	28.07 ± 0.75	0.0009	0.22 ± 0.19
PaLACS2	28.75 ± 0.82	30.08 ± 1.13	0.0002	0.13 ± 0.06
PaLTPG1	23.61 ± 1.31	24.67 ± 1.55	0.0003	0.14 ± 0.15
PaPIP1;4	15.63 ± 0.24	20.3 ± 1.29	0.1083	1.22 ± 0.21
PaWINA	30.2 ± 0.58	29.72 ± 1.65	0.0001	0.04 ± 0.01
PaWINB	27.9 ± 0.54	28.65 ± 0.98	0.0001	0.09 ± 0.04
PaWS	21.99 ± 1.43	26.63 ± 1.64	0.7048	1.56 ± 1.15

Raw Ct mean and standard deviation values. Additionally adjusted p-value from Wilcoxon rank sum test is provided together with mean and standard deviation of expression fold changes (2−^ΔΔCt^). P values below 0.05 are statistically significant. Mean Fold Changes and Standard deviation are also calculated.

**Table 2:** Quantification results of dPCR in high and low CI samples.

Genes	Mean copies ± SD High CI	Mean copies ± SD Low CI	*P*-value adjusted	Mean FC ± SD
PaAct	28,159 ± 12,957	2517 ± 2945	NA	NA
PaB-Gal	385 ± 189	50 ± 54	0.4384	0.47 ± 0.19
PaCer1	1663 ± 842	47 ± 43	0.0447	2.14 ± 1.24
PaCer3	30 ± 15	10 ± 5	0.0095	0.1 ± 0.06
PaCYP79A9	244 ± 186	17 ± 8	0.7802	0.39 ± 0.27
PaEG	592 ± 541	60 ± 68	0.3237	0.47 ± 0.6
PaExp1	14,293 ± 10,414	1836 ± 1013	0.0391	0.39 ± 0.34
PaExp2	19,434 ± 10,660	4945 ± 2957	0.0008	0.16 ± 0.08
PaKCR1	2262 ± 2041	242 ± 190	0.2275	0.7 ± 0.73
PaKCS6	52 ± 51	18 ± 14	0.0095	0.09 ± 0.1
PaLACS2	470 ± 134	47 ± 33	0.5677	0.52 ± 0.16
PaLTPG1	3792 ± 1744	319 ± 322	0.7048	0.81 ± 0.5
PaPIP1;4	79,762 ± 41,490	4649 ± 6356	0.0391	1.05 ± 0.29
PaWINA	12 ± 6	11 ± 3	0.0003	0.04 ± 0.03
PaWINB	24 ± 16	25 ± 22	0.0006	0.03 ± 0.03
PaWS	4274 ± 3400	24 ± 13	0.0005	7.25 ± 4.79
PaXTH	86,702 ± 64,774	402 ± 671	0.0003	19.26 ± 14.32

Raw mean total copy number and standard deviation. Additionally adjusted p-value from Wilcoxon rank sum test is provided together with mean and standard deviation of expression fold changes (expression ratios). P values below 0.05 are statistically significant. Mean Fold Changes and Standard deviation are also calculated.

**Fig. 2:**
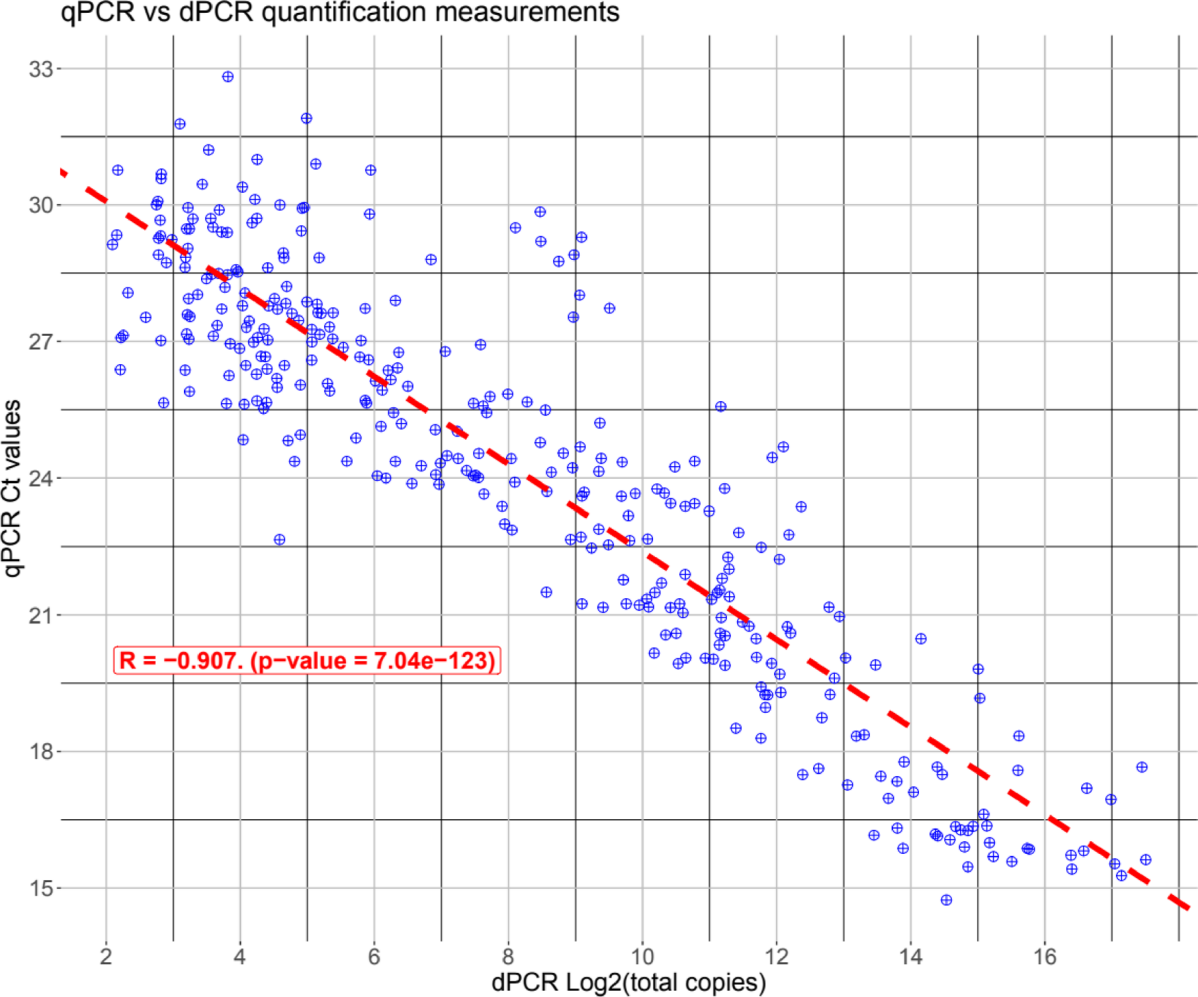
Plot of log_2_ transformed DNA copies obtained from dPCR vs. qPCR.

To test the degree of correspondence between qPCR and dPCR data, we performed a correlation between qPCR and dPCR data. We previously performed a log_2_ transformation of the dPCR count data to make them logarithmic as the Ct values are (Fig. 2). As expected, we found a negative correlation with a Pearson R coefficient of −0.907 with a p-value of 7.04e-123. This high correlation indicates that dPCR and qPCR are giving similar results.

Given the linear relationship, the equation that can be used to transform qPCR data into DNA reads is:

Cts = 32.0026–0.9621*Log2Copies.

This equation has a degree of error of 0.093, thus it may give an estimation of the reads and help compare between experiments in a more feasible way (see discussion).

### Differences in gene expression using qPCR vs. dPCR

**Fig. 3:**
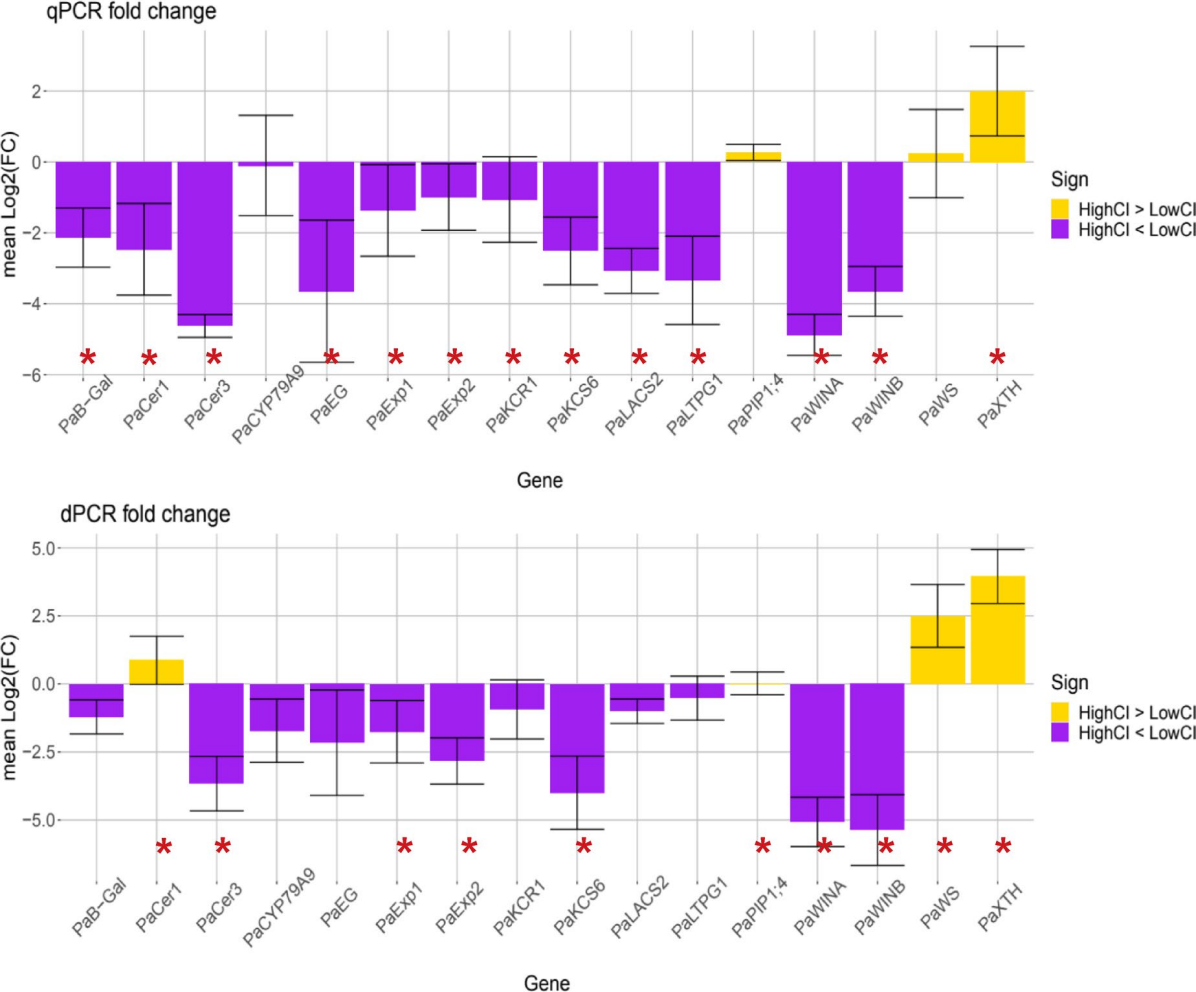
Relative gene expression by qPCR using ∆∆Ct method, and relative transcripts number by dPCR. Transcripts number were determined as a fold change using cherries with low CI as a control. Each bar represents the mean ± SD.

We compared the results given by qPCR and dPCR technologies for a set of 16 genes. After the p-value adjustment, the qPCR data showed significant differences between CI groups for 13 genes while the dPCR indicated that there were 10 (See Tables 1 and 2; Fig. 3). A total of eight genes were differentially expressed according to both PCR technologies. These were *PaXTH*,* PaWINA*,* PaWINB*,* PaKCS6*,* PaCER3*,* PaCER1*,* PaEXP2* and *PaEXP1.* When measured with dPCR even though the fold change in expression is minimal (1.05 and 0.02 in logarithmic scale) the Wilcoxon rank sum test turned out significant for *PaPIP1;4*. This is due to an outlier sample in the low CI group with an extreme value of referenced copies of 12.81 compared to the mean of this group of 2.67. This outlier changed the probability distribution for this gene and group enough to make the Wilcoxon rank sum test significant when compared with the distribution of samples with high CI. Thus, even if the test was significant, *PaPIP1;4* is unlikely to be differentially expressed. We interpret that this discrepancy is due to the higher variability in gene expression quantification obtained with dPCR compared to qPCR Ct values.

The fold changes in expression using the qPCR data showed that all but three genes, *PaPIP1;4*,* PaWS* and *PaXTH*, were down-regulated in high CI samples compared to low CI. The result for the dPCR is very similar as it agrees with qPCR on the fold change sign for 15 out of 16 genes. There is but one discrepancy for the gene *PaCER1* that had the opposite fold change sign and is up-regulated in high CI samples. The highest fold changes with negative signs corresponded to the genes *PaWINA* and *PaWINB* according to qPCR and dPCR data respectively. The highest positive fold changes were registered for *PaXTH* with both PCR methodologies. In summary, qPCR and dPCR gave similar results regarding the assessment of differential gene expression.

### Integrative analysis of cracking-related genes in sweet Cherry

**Fig. 4:**
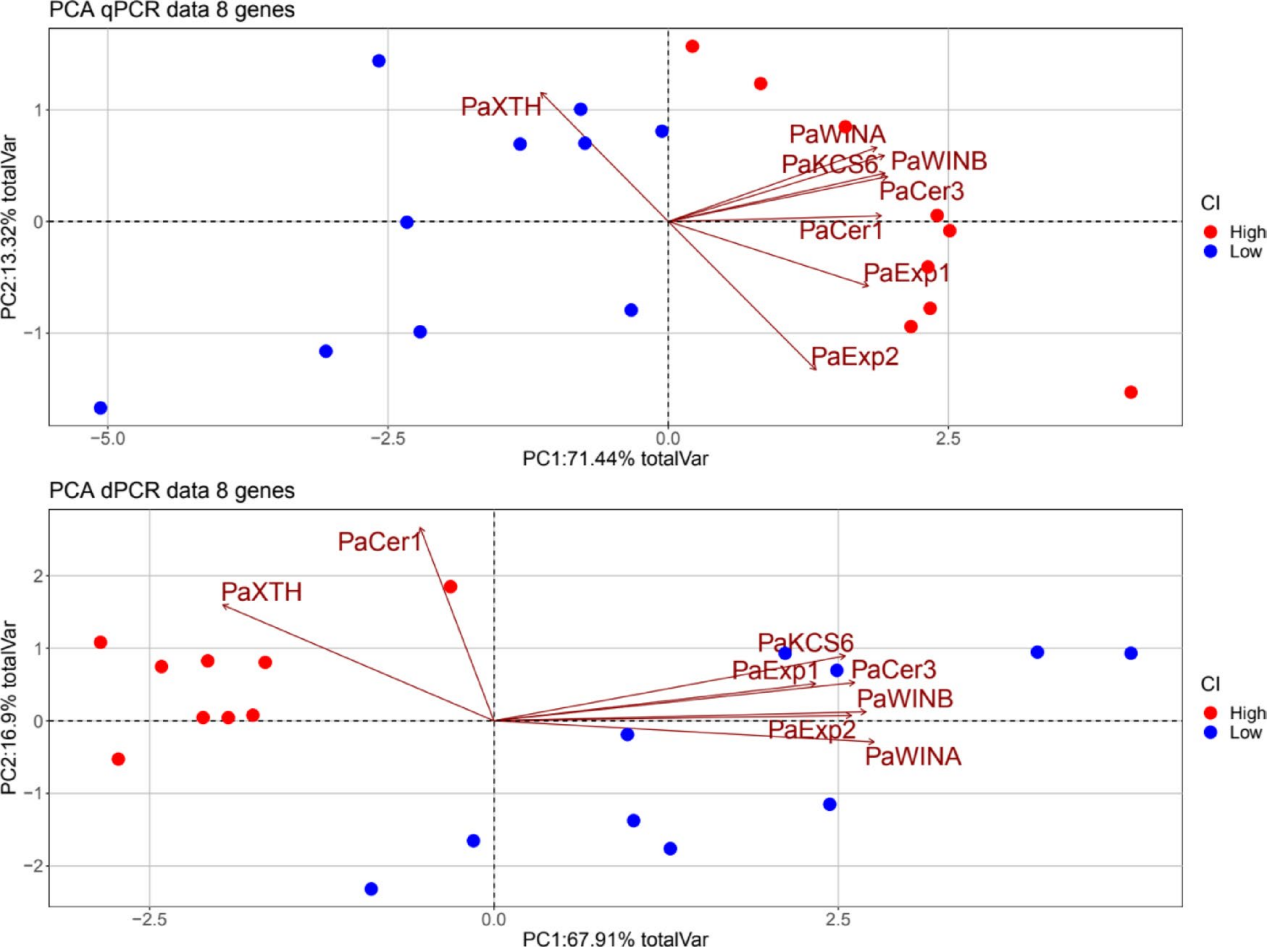
PCA-Biplot of samples with Low CI and samples with High CI based on gene expression by qPCR, and on transcripts number by dPCR. We used data from cracking-related genes with significative differences between the two contrasting cracking levels according Wilcoxon rank sum test. The genes used are *PaExp1*, *PaExp2*, *PaXTH*, *PaKCS6*, *PaCer1*, *PaCer3*, *PaWINA*, and *PaWINB*. Points refer to the samples and vectors refer to the genes.

We selected eight genes with significant differential expression according to both PCR technologies. We performed a PCA analysis (Fig. 4). The first two PC explained 84.76% of the total variance of the qPCR data. In the case of dPCR data, the first two components explained an almost identical 84.81% of the total variance. Furthermore, in both cases, the samples could be visually clustered together according to their CI group. Thus, dPCR output data contains equivalent information to that of qPCR. The genes selected showed predictive capacity of CI based on their expression values. The contribution to the PCs was slightly different for each PCR technology. In the case of qPCR, *PaXTH* and *PaEXP2* contributed approximately equally to PC1 and PC2 while the remaining genes were mostly the base of PC1. PC1 alone in the qPCR case is capable to do a flawless classification of the samples. For the dPCR data, *PaXTH* kept the same tendency and contributed to both PC1 and PC2 while *PaCER1* contributed mostly to PC2 alone. The other genes contributed to PC1. This time, PC1 by itself was not enough to accurately classify all samples, needing the information of PC2.

**Fig. 5:**
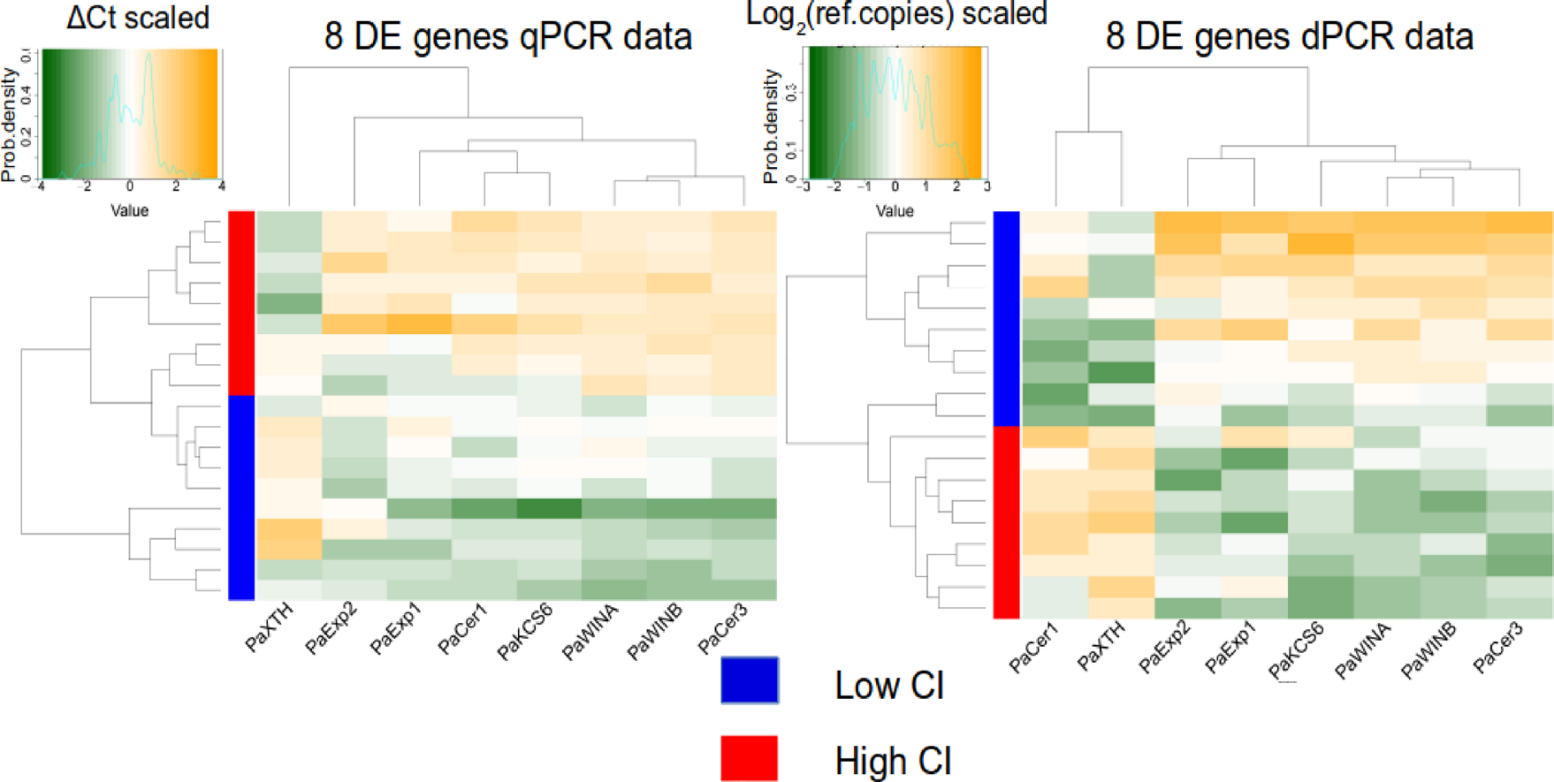
Clustering heatmap of samples with Low CI and samples with High CI based on gene expression by qPCR, and on transcripts number by dPCR, using data from cracking-related genes with significative differences between the two contrasting cracking levels according Wilcoxon rank sum test, namely *PaExp1*, *PaExp2*, *PaXTH*, *PaKCS6*, *PaCer1*, *PaCer3*, *PaWINA*, and *PaWINB* genes.

Finally, we visualized the clustering results with a heatmap (Fig. 5). As was the case with the PCA, a clear separation of samples based on their CI was achieved with both PCR technologies. The genes were grouped in two clusters according to their expression fold change signature (see Fig. 5) with both PCR technologies. The interpretation of dPCR data is easier because a higher value of copies in the reaction is indicative of higher expression. In the case of qPCR, this relationship is inverse i.e. a higher Ct value is indicative of lower expression. Thus, in Fig. 4 the position of the rows, that is, the samples, is inverted between each PCR result. In summary, dPCR data allows to achieve an equivalent visualization of the dataset while being easier to comprehend than qPCR data. Our results show the potential value of this set of genes to be used as an expression bitmap marker system for fruit cracking.

## Discussion

Fruit cracking is a common agricultural issue that affects the quality, yield, and marketability of fruits. It occurs when the outer skin of the fruit splits, usually during the ripening process. The phenomenon is caused by a combination of environmental, physiological, and genetic factors^2,23^. Our previous works have demonstrated that the application of different compounds in the field reduced the cracking index in sweet cherries. Specifically, a combination of calcium and abscisic acid, and calcium and glycine betaine significantly reduced cracking in cv. Sweetheart^24^. Similarly, foliar application of calcium and *Ascophyllum nodosum* at different concentrations resulted in lower CI in cv. Burlat^4^.

In this work we have performed a direct comparison of qPCR and dPCR as a mean to study gene expression in cherry fruits. It does not come as a surprise that when paired samples are analyzed the correlation between both techniques is high. While transcriptomic data are usually normalized for gene length, and number of reads per kilobase per million mapped reads (RPKM)^25^dPCR gives as an initial outputs the total number of raw reads for a given gene. Gene length does not play a role in PCR normalization techniques. However GC content has an impact on PCR and is a parameter taken into account during primer design^19^. Thus it is a within-sample effect in transcriptomics when comparing genes in the same sample^26^. In this respect, qPCR has the built in advantage that PCR efficiency is an output of the reaction measuring GC content in an indirect way for comparison within samples. However, we consider that in the long run, dPCR may be a better technique. Indeed, relating the number of reads to transcriptomics may be easier than using qPCR data. Nevertheless, the correlation equation described in this work may be useful to obtain an estimation of read numbers in qPCR without doing a dilution curve for every gene used.

We have confirmed the results previously described in the literature for the genes we tested. dPCR is prone to give higher variability in the results compared to qPCR but the measured quantity of total copies in the reaction is more readily understood than Ct values. The general tendency of the genes selected in this work is to be down-regulated in high CI fruits except for *PaWS* and *PaXTH.* Differential expression of *PaXTH* has been found in different experiments in cherry during storage^27,28^ and our results confirm its differential expression in cracking.

The differentially expressed bitmap identified is limited in size. However, it points towards several biological functions defined as Gene Ontology terms.

Our work describes correlation in gene expression with CI but the functional analysis in *Prunus* is missing. As the biological pathways identified by the GO terms associated are relevant to fruit cracking, they may be good candidates for target modifications.

We conclude that dPCR is a method that gives comparable results of those obtained with qPCR. dPCR has as major advantage the output as number of reads. This allows comparing between experiments with ease and helps integrate transcriptomic studies. In contrast the standard qPCR has a larger range of gene expression that can be measured on a single plate and consumables are more affordable than those of dPCR.

## Materials and methods

### Plant material

**Fig. 6:**
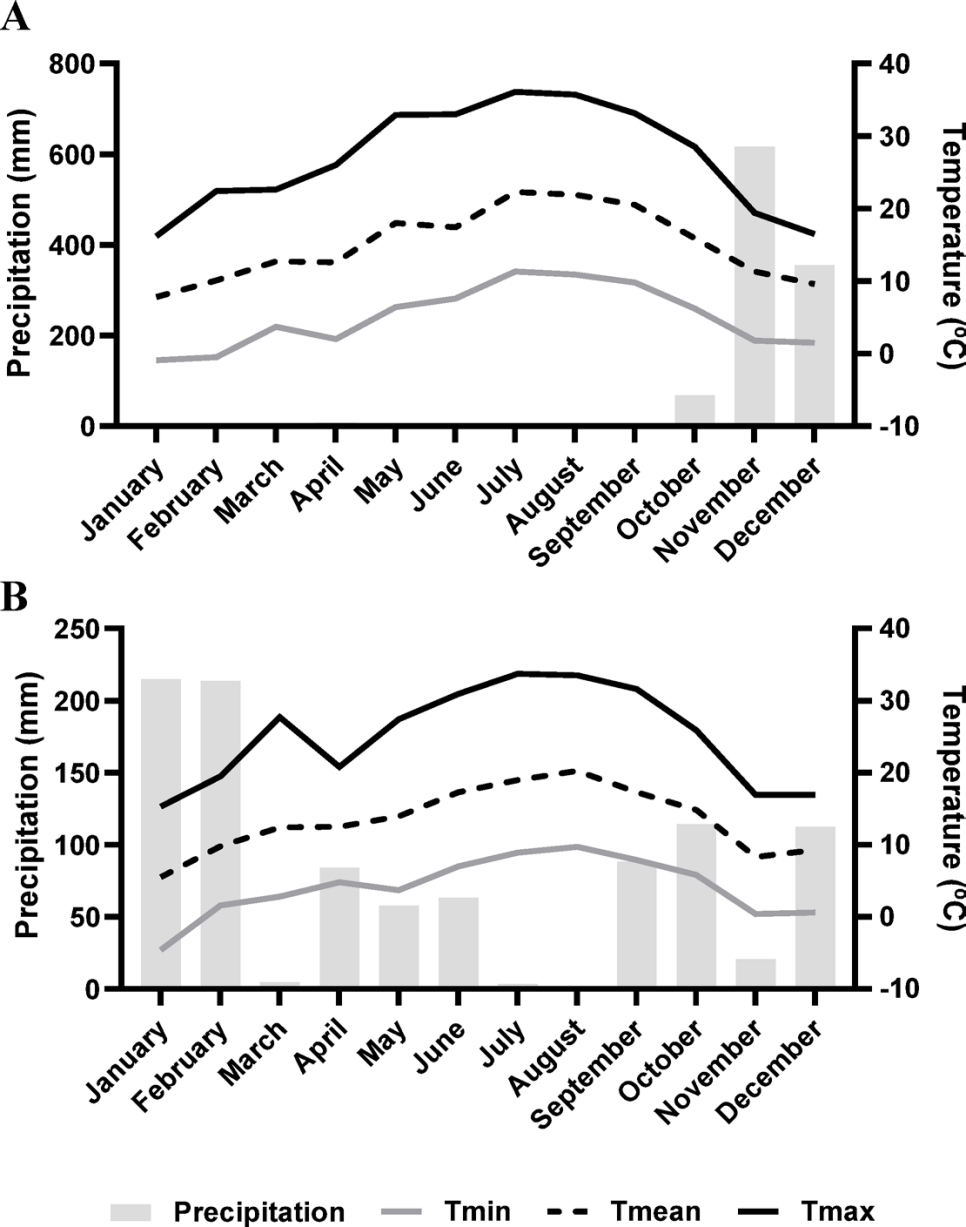
Weather conditions near the orchard of cv. Burlat (**A**), and the orchard of cv. Sweetheart (**B**). Climatic conditions refer to precipitation (mm), minimum, mean and maximum temperatures (ºC).

Experiments were carried out in commercial orchards located in Resende region, Portugal (41°06’52.8"N 7°56’13.3"W, altitude 246 m – Cv. Burlat, and 41°04’55.3"N 7°53’35.2"W, altitude 615 m – Cv. Sweetheart). The weather conditions, namely precipitation and temperatures (minimum, mean, and maximum), were recorded in weather stations near the orchards (Fig. 6). Temperatures were similar in both orchards. In contrast, precipitation highly varied between the orchards, with high rain levels in the orchard of cv. Sweetheart.

Fruits were collected at their ripening stage, that is, the optimal time for consuming, and the cracking index (CI) was determined. CI is defined as the percentage of cracked fruits, and it is given by the following formula: CI = $\:\frac{\left(5a+3b+c\right)*100}{250}$, where *a*, *b* and *c* correspond to the number of cracked fruits after 2, 4 and 6 h immersion in distilled water^29^. For each cultivar, 15 sets of 50 healthy fruits were selected to achieve the CI according to the formula above. To perform this work, we used healthy sweet cherries with different cracking susceptibilities from a highly susceptible cultivar, Burlat (CI higher than 60%) and a moderate susceptible cultivar, Sweetheart (CI lower than 30%). From here on, we will refer to the Burlat samples as having a high CI and the Sweetheart samples as having a low CI.

### RNA extraction and cDNA synthesis

Total RNA was isolated from whole fruit exocarp from fruits with different cracking susceptibilities. We extracted 19 samples (each sample corresponds to one healthy cherry), of which 10 fruits were from Cv. Burlat and 9 from Cv. Sweetheart. RNA was extracted using the GF-1 Total RNA Extraction Kit (Vivantis, Malasya) according to manufacturer’s instructions, followed by an electrophoresis in 10 g L^−1^ agarose gel (80 V for 90 min) to evaluate the RNA integrity. Additionally, RNA concentration and purity (A260/A280 ratio) was accessed in Biotek PowerWave XS Microplate Reader (Marshall Scientific, Hampton, United Sates), coupled with the gen5 program. Then, RNA samples were diluted to 100 ng µL^−1^ and reverse transcribed to cDNA using the High-Capacity cDNA Reverse Transcription Kit (Applied Biosystems^™^, Thermo Fisher Scientific, Vilnius, Lithuania), also according to manufacturer’s instructions, and kept at − 20 °C until further analysis.

### Quantitative real-time polymerase chain reaction (qPCR) analysis

The expression of sweet cherry cracking-related genes was evaluated by quantitative real-time PCR (qPCR). We used a set of genes described as differentially expressed between low and high CI in sweet cherries. These included *PaEXP1*,* Paβ-GAL*,* PaWS*,* PaKCS6*,* PaLTPG1*,* PaWINB*,* PaACT*^22^ *PaPIP1;4*^30^, *PaXTH*,* PaEG*^27^, *PaKCR1*, * PaCER1*, * PaCER3*, * PaWINA*^31^ and *PaLCAS2*^32^. We also designed additional primers to amplify *PaEXP2 (*NCBI Accession number AF297522.1) and *PaCYP78A9* (NCBI Accession number XM_021959332.1) (Supplementary table S1). We used *PaACT* (actin) as a housekeeping gene for normalization^22^. Actin was previously described as the most stable reference gene in sweet cherry (Ye et al., 2015). Each qPCR reaction mixture had a final volume of 10 µL constituted by 5 µL of SYBR™ Select Master Mix (Applied Biosystems™, Thermo Fisher Scientific, Vilnius, Lithuania), 1.5 µL of the cDNA template, 0.4 µL of forward primer (10 µM), 0.4 µL of reverse primer (10 µM), and 2.7 µL of distilled water. The reactions were performed in the StepOnePlus™ Real-Time PCR system (Applied Biosystems™, Thermo Fisher Scientific, Foster City, USA) using the following program: UDG activation for 2 min at 50 ºC, initial denaturation for 5 min at 95 °C, followed by 40 cycles of 10 s at 95 °C, 20 s at Ta and 15 s at 72 °C, and a melting curve of 15 s at 95 °C, 1 min at 60 ºC and 15 s at 95 °C, to exclude interference of primer dimers, and other non-specific products. We analysed the qPCR melting point of all the genes analysed and verified that they had a single melting peak (Supplementary Fig. 1). We used two technical replicates per sample. A negative water control was included on each plate to check the cDNA contaminants and validate the results. The threshold quantification cycle (Ct) values were accessed using the StepOne™ software v2.3 (Thermo Fisher Scientific), leading to a Ct mean value of the two technical replicates, where only a standard deviation (SD) below 0.5 was considered. The relative quantification of each target gene was normalized by the comparison to the housekeeping gene^16^ followed by the analysis of the relative gene expression using the 2^−ΔΔCt^ method^33^. Results are expressed as the average of the replicates within each group and its SD.

### Digital polymerase chain reaction (dPCR) analysis

The transcripts number of sweet cherry cracking-related genes was evaluated by digital PCR (dPCR), using the same sweet cherry mRNA samples that were used in qPCR experiments. Likewise, we used the same set of primers for each gene (Table S1), as well as *PaACT* gene as reference gene^22^. dPCR reactions consisted of 4 µL of QIAcuity^®^ EvaGreen PCR Mastermix (QIAGEN, Hilden, Germany), 1.5 µL of cDNA template, 0.5 µL of forward primer (10 µM), 0.5 µL of reverse primer (10 µM), and 5.5 µL of distilled water for a total of 12µL. The reaction mixtures were transferred to the QIAcuity nanoplates 8.5 K 96-well, and performed in the QIAcuity Digital PCR System (QIAGEN, Hilden, Germany), according the dPCR default parameters. First, a QIAGEN standard priming profile step was completed to seal the partitions in the nanoplate, followed by the thermocycling step to perform the polymerase chain reaction using the following program: initial denaturation for 2 min at 95 °C, followed by 40 cycles of 15 s at 95 °C, 15 s at Ta and 20 s at 72 °C, and a final extension of 2 min at 72 ºC. Lastly, the imaging step, in the green channel with an exposure duration of 400 ms and a gain of 4, allowed the image acquisition of all wells to check the fluorescent light for further analysis. As in qPCR, two technical replicates per sample and a negative water control was included on each nanoplate to check the cDNA contaminants and validate the results. The absolute transcripts quantification, in copies/µL, was accessed using the QIAcuity Software Suite 2.1.7.182 (QIAGEN, Hilden, Germany), leading to a mean of copies/µL of the two technical replicates in each sample. The absolute quantification was only validated if at least one third of total valid partitions were negative. Thus, cDNA was diluted 1:10 to analyse *PaACT*, *PaEXP1*, *PaEXP2*, and *PaPIP1;4*. Normalization was achieved by dividing the number of reads of a target gene by the number of reads of the reference gene. Fold changes between high and low CI were obtained by dividing the normalized data of high CI by the low CI. For each target gene, results are expressed as the average of the replicates within each group and its SD.

### Statistical analysis

We used Software R version 4.2.3 (The R Foundation for Statistical Computing, Vienna, Austria) to perform the analysis.

We performed a global correlation analysis between dPCR and qPCR data using the Pearson method. We log_2_-transformed the dPCR data prior to this analysis as well. The Pearson correlation coefficient R was tested for being significantly different from 0 with a T-test.

Every gene was tested for differential expression between high and low CI samples using the Wilcoxon rank sum test. We tested every gene once with qPCR data and then with dPCR data. P-value adjustment was applied to all tests using the Benjamini-Hochberg False Discovery Rate (FDR) method. Significant genes (with an adjusted p-value lower than 0.05) according to the data originating from both qPCR and dPCR were selected for further analysis. To perform the statistical tests, we used normalized expression values. For qPCR we made use of the ∆Ct values and for dPCR we employed the referenced copy number (target gene copies divided by reference gene copies).

To obtain the fold changes in expression we first calculated the mean expression across every control sample for every gene. We designated the samples of low CI as the control. Then we applied the ∆∆Ct method^33^ in the case of qPCR data for every gene and sample of high CI. Similarly, in the case of dPCR, for every gene and high CI samples we calculated the ratio of expression to the mean of low CI. Average fold changes according to qPCR and dPCR were obtained for every gene and log_2_-transformed for visualization purposes.

We used the principal component analysis (PCA) dimensionality reduction method to visualize the dataset using the selected differentially expressed genes. We compared the result of using qPCR or dPCR. Prior to the PCA, dPCR data was log_2_ transformed to convert it to a comparable scale of that of Ct values. Both ∆Ct and referenced copies data were centered and scaled before conducting the PCA. We used the first two components to plot the samples in a scatter-plot. We also plotted the contribution of each variable/gene to the principal components (PCs) with arrows. The cosine of these arrows to the axes is directly proportional to their contributions to the PCs. Lastly, as a complementary visualization to the PCA, a hierarchical clustering analysis was performed using the Euclidean distance between samples and the complete link function. Similarly, as before, we compared the result using qPCR and dPCR data. The data was transformed in the same way as described for the PCA prior to this analysis. The result was visualized with a heatmap.

## Electronic supplementary material

Below is the link to the electronic supplementary material.

Supplementary Material 1

Supplementary Material 2

Supplementary Material 3

## Data Availability

The datasets generated and/or analysed during the current study are available in the ZENODO repository, 10.5281/zenodo.14591893.
