# Supplementary material for: Validation of a bitmap of genes involved in cherry fruit cracking by digital PCR and qPCR, suitable for plant breeding
Source: Sci Rep. 2025 Jul 22;15:26619. doi: 10.1038/s41598-025-11006-w (PMC12284209; doi:10.1038/s41598-025-11006-w)
Supplement: Supplementary file 1 — Supplementary Material 1 [file 41598_2025_11006_MOESM1_ESM.docx]

Supplementary information

Dos Santos et al

**Table S1.** Primer sequences (5’🡪 3’), annealing temperature (Ta) and amplicon size (bp) used for quantitative real-time PCR and digital PCR reactions.

| Gene |  | Sequence (5' 🡪 3') | Ta (ºC) | Amplicon size (bp) | Reference |
| --- | --- | --- | --- | --- | --- |
| *PaEXP1* | *Forward* | GCTCAGTCCAACGATAATGG | 57 | 113 | Balbontín et al. (2014) |
|  | *Reverse* | GTAACAGGCACAATCCCAG |  |  |  |
| *PaEXP2* | *Forward* | GTCTTCCAGCACATTGCTCA | 57 | 185 | This study |
|  | *Reverse* | GTCCTGGACCCTTTGACTGA |  |  |  |
| *PaXTH* | *Forward* | GCTTGTGAAGGCAGATTGGAG | 57 | 86 | Belge et al. (2017) |
|  | *Reverse* | AGCCTGAAGACCAAGTGCAAG |  |  |  |
| *Paβ-Gal* | *Forward* | ATGCCAAGGTTTCTGTGGAG | 57 | 103 | Balbontín et al. (2014) |
|  | *Reverse* | GCTGTACGGGACCAGTGATT |  |  |  |
| *PaEG* | *Forward* | GATTGAGAATGCCAAGGATGC | 57 | 103 | Belge et al. (2017) |
|  | *Reverse* | GTTTGGATCTGCCACTTGGAC |  |  |  |
| *PaCYP78A9* | *Forward* | CTTCCATGGTTGGCTGATTT | 57 | 165 | This study |
|  | *Reverse* | CGAAAGCAAGACGTCAACAA |  |  |  |
| *PaWS* | *Forward* | TCTCAACCACCAAACACAGC | 57 | 102 | Balbontín et al. (2014) |
|  | *Reverse* | CCTTCATAGTGTCGGCGATT |  |  |  |
| *PaKCS6* | *Forward* | AGGAAGAGCAACTGCTCCGA | 60 | 90 | Alkio et al. (2012); Balbontín et al. (2014) |
|  | *Reverse* | TCTGATGGCGATCGCAGGAG |  |  |  |
| *PaKCR1* | *Forward* | GGGCTCAATCTGGTCTTGG | 60 | 160 | Alkio et al. (2012) |
|  | *Reverse* | CTTCAATGGTCTCACGAATGC |  |  |  |
| *PaLTPG1* | *Forward* | ATGAGGAAGATGTTGGTGGTG | 60 | 97 | Alkio et al. (2012); Balbontín et al. (2014) |
|  | *Reverse* | ACTGGTTGCTACACTTCTG |  |  |  |
| *PaCER1* | *Forward* | TCCTCTACTCTCGCTACCATT | 57 | 137 | Alkio et al. (2012) |
|  | *Reverse* | CGTGAATATTGTCGTCAACATAGG |  |  |  |
| *PaCER3* | *Forward* | GTCACAAAGTACCAAGCTGCCCG | 63 | 181 | Alkio et al. (2012) |
|  | *Reverse* | CATCAGGTAGTCTCATGGCTGCA |  |  |  |
| *PaLACS2* | *Forward* | ATACTGTTGTCTGGTGCTG | 57 | 198 | Alkio et al. (2012); Declercq et al. (2014) |
|  | *Reverse* | CACTGACTCAAGCCTTGT |  |  |  |
| *PaWINA* | *Forward* | AGCCCGAGCCTACGATGAAGCA | 67 | 166 | Alkio et al. (2012) |
|  | *Reverse* | CGAATGGTCCAAGTCGCTCGAA |  |  |  |
| *PaWINB* | *Forward* | AATGGTGGGATTGATTGAGG | 57 | 119 | Alkio et al. (2012); Balbontín et al. (2014) |
|  | *Reverse* | TTGTTGGTGAAGGAATTGGA |  |  |  |
| *PaPIP1;4* | *Forward* | cgtgtacttatcaagctctctcaag | 57 | 128 | Chen et al. (2019) |
|  | *Reverse* | ttgctgctcattaacactggct |  |  |  |
| *PaAct* | *Forward* | TGAAGATTAAGGTTGTGGCTC | 57 | 114 | Balbontín et al. (2014) |
|  | *Reverse* | CGTACTCACCCTTGGAAATC |  |  |  |
