# Supplementary material for: Validation of a bitmap of genes involved in cherry fruit cracking by digital PCR and qPCR, suitable for plant breeding
Source: Sci Rep. 2025 Jul 22;15:26619. doi: 10.1038/s41598-025-11006-w (PMC12284209; doi:10.1038/s41598-025-11006-w)
Supplement: Supplementary file 2 — Supplementary Material 2 [file 41598_2025_11006_MOESM2_ESM.pdf]

Supplementary figure 1

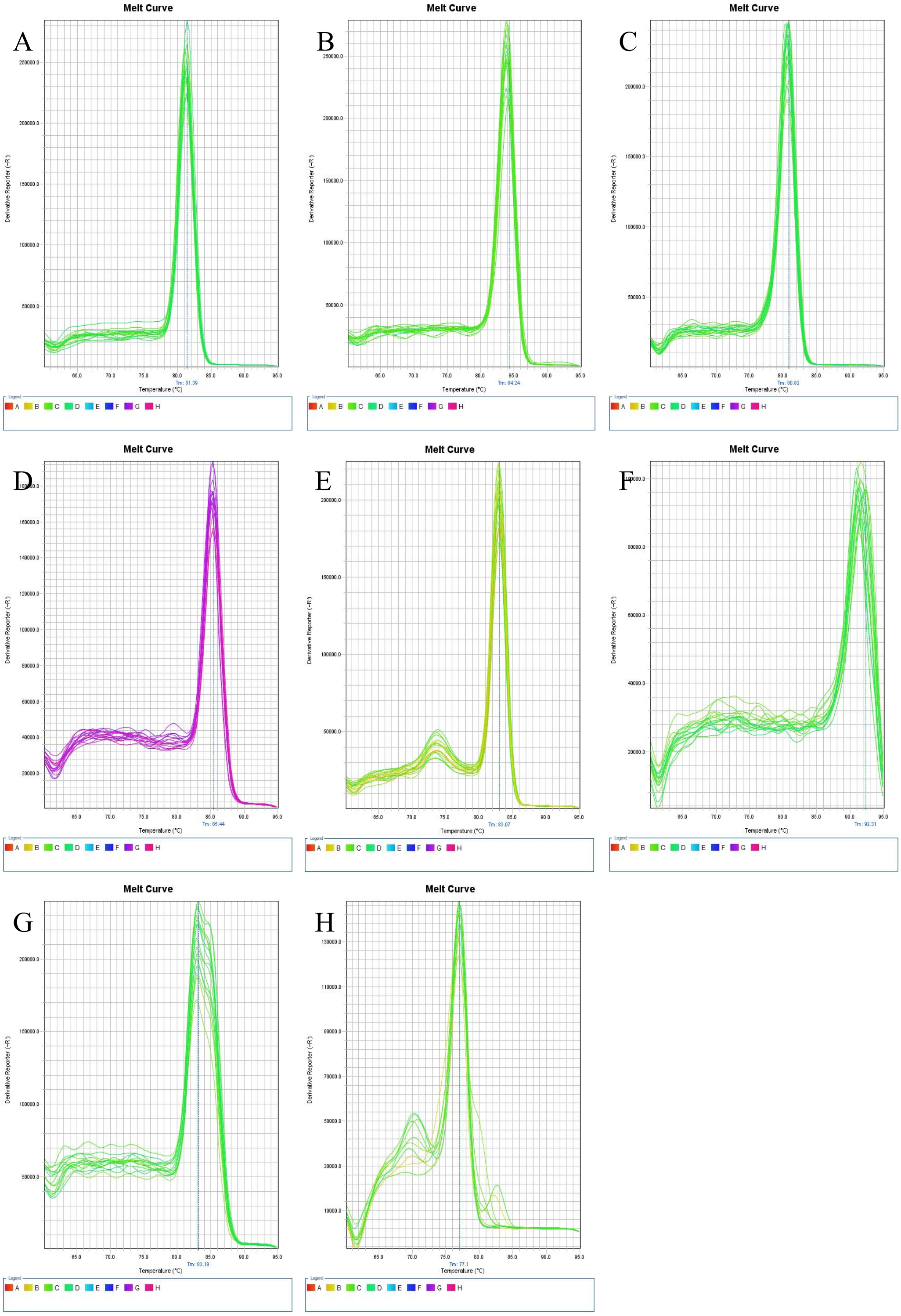

Melting curves for genes (A) *PaAct*, (B) *PaExp1*, (C) *Paβ-Gal*, (D) *PaKCS6*, (E) *PaKCR1*, (F) *PaCer3*, (G) *PaWINA*, and (H) *PaWINB*.
