# Supplementary figures and images for: Validation of a bitmap of genes involved in cherry fruit cracking by digital PCR and qPCR, suitable for plant breeding
Source: Sci Rep. 2025 Jul 22;15:26619. doi: 10.1038/s41598-025-11006-w (PMC12284209; doi:10.1038/s41598-025-11006-w)

Supplementary Figure 2

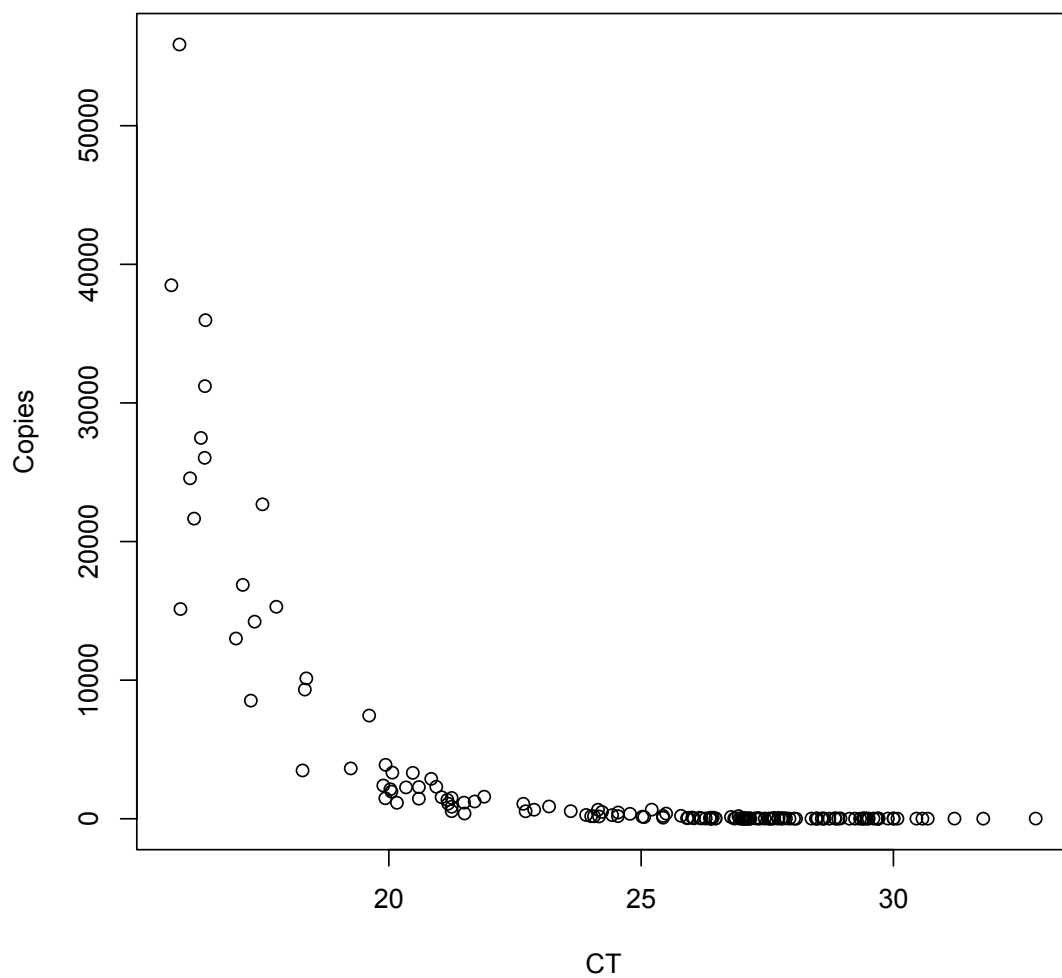

### Plotting of qPCR CT values versus dPCR copies

Supplement: Supplementary file 3 — Supplementary Material 3 [file 41598_2025_11006_MOESM3_ESM.pdf]
